# Development of a scale to measure reasons for eating less healthily after exercise: the compensatory unhealthy eating scale

**DOI:** 10.1080/21642850.2020.1734007

**Published:** 2020-02-27

**Authors:** Natalie M. Reily, Lenny R. Vartanian, Kate Faasse

**Affiliations:** School of Psychology, UNSW Sydney, Sydney, Australia

**Keywords:** Compensatory eating, scale development, exercise, weight loss

## Abstract

Objective: Patterns of ‘compensatory eating’ following exercise are likely to be harmful for long-term health and counterproductive for weight loss goals. However, little is known about reasons why people eat unhealthily after exercising. Thus, we aimed to develop a measure that assesses reasons why people engage in compensatory unhealthy eating. Method: A multi-stage approach using exploratory and confirmatory factor analysis was used to develop and replicate a scale and validate its psychometric properties in three different samples. Participants (total *N* = 814) rated their agreement with statements capturing different reasons for eating less healthily after exercise. Results: Factor analysis revealed four distinct factors underlying compensatory eating: Reward for Effort, Permission to Consume, Need to Consume, and Reduced Self-Control. The resulting Compensatory Unhealthy Eating Scale (CUES) had good internal consistency and convergent validity. Conclusion: The CUES has utility as a tool to assess compensatory eating behaviour. Further research should examine who is most likely to compensate and under what circumstances. Broadening current knowledge of compensatory eating after exercise may facilitate development of strategies to improve health behaviour regulation.

Many people begin an exercise programme with the primary goal of losing weight. Although regular exercise results in numerous health benefits irrespective of whether or not people do lose weight, some people become disillusioned with exercise because they are unsatisfied with the amount of weight that they lose (Shaw, Gennat, O’Rourke, & Del Mar, 2006). The average amount of weight that people lose with programmes that target exercise is modest at best, and is significantly less than would be expected based on the magnitude of the energy deficit (Donnelly & Smith, 2005; Johns, Hartmann-Boyce, Jebb, & Aveyard, 2014; Ross & Janssen, 2001; Shaw et al., 2006).

One obvious reason why some people might not be losing as much weight as expected in intervention studies is noncompliance; that is, they may not actually be doing the prescribed exercise (Melanson, Keadle, Donnelly, Braun, & King, 2013). However, lack of compliance to programmes alone cannot explain the variability in amount of weight lost, because lower-than-expected weight loss can be observed even when exercise is monitored throughout these intervention studies (Donnelly et al., 2003; King, Hopkins, Caudwell, Stubbs, & Blundell, 2009). Another reason that weight loss might be lower than expected or desired is that some people who start exercising might also compensate for the exercise by engaging in other behaviours that make losing weight more difficult, such as increasing their food intake or eating less healthily after having exercised (King et al., 2007; Melanson et al., 2013). Indeed, one study found that 75% of people reported eating more on exercise days at least sometimes (Moshier et al., 2016). Furthermore, exercise has been associated with increased preference for hedonic foods (Finlayson, Bryant, Blundell, & King, 2009), increased approach motivation for unhealthy foods (May, Nock, Bentley, & Demaree, 2018), and changes to food palatability (Elder & Roberts, 2007). Thus, compensatory eating might underlie some of the variability observed in weight-loss responses to exercise, and also has implications for long-term wellbeing irrespective of how much it influences weight. It remains unclear, however, why exactly compensatory eating occurs and who is most susceptible to this behaviour.

Experimental research suggests that psychological factors are likely to play a role in compensatory eating over and above any purely physiological effects (e.g. changes in appetite and satiety, gut physiology and responsiveness and metabolic rate; for reviews see King et al., 2007; Melanson et al., 2013). For example, Werle, Wansink, and Payne (2015) had participants take a 30-minute walk around a university campus prior to having a buffet lunch, and the walk was framed as either an ‘exercise’ activity or a ‘fun’ activity. Participants in the ‘exercise’ co­ndition consumed more calories of hedonic foods (Study 1) and served themselves more calories (Study 2) than did those in the ‘fun’ condition. In another study, participants exercised in the laboratory on a stationary bike until they had all expended approximately 120 kcal (McCaig, Hawkins, & Rogers, 2016). After exercising, participants were (falsely) informed that they had burned either 50 kcal or 265 kcal, and were then given access to food to sample during a taste test. The ‘265 kcal’ group consumed more food than did the ‘50 kcal’ group, and this difference was driven by greater intake of hedonic foods (i.e. cookies). In both the Werle et al. study and the McCaig et al. study, there was no group difference in participants’ actual energy expenditure, but their perceptions of the number of calories expended during exercise influenced their subsequent food intake. Together, these studies suggest that psychological or motivational factors are relevant for understanding compensatory eating after exercise.

One way that psychological factors may be involved in compensatory eating after exercise is that people may hold ‘compensatory health beliefs,’ which are notions that certain unhealthy behaviours (e.g. eating unhealthily) can be ‘neutralised’ or compensated for by engaging in subsequent healthy behaviours (e.g. going to the gym; Knäuper, Rabiau, Cohen, & Patriciu, 2004; Rabiau, Knäuper, & Miquelon, 2006). The Compensatory Health Beliefs Scale (CHBS) was developed as a broad measure to assess the extent to which people endorse beliefs about the interchangeability of different kinds of health behaviours across a number of health domains, including stress, substance use, weight control and sleep hygiene (Knäuper et al., 2004). People who endorse these general beliefs might permit themselves to indulge in unhealthy behaviours because planning to compensate for an unhealthy behaviour later absolves them of the guilt they would otherwise feel about engaging in the unhealthy behaviour. In line with this idea, there is a positive association between endorsing compensatory beliefs and engaging in unhealthy behaviours (Kronick & Knäuper, 2010; Kronick, Auerbach, Stich, & Knäuper, 2011). These types of general compensatory beliefs might also apply to the more specific context of compensatory eating following exercise.

One existing measure that examines reasons for eating post-exercise is the Compensatory Eating Motivations Questionnaire (CEMQ; Moshier et al., 2016). This measure assesses various motivations that people have for eating after exercising, including recovery (e.g. ‘Eating returns my body to normal’), relief (e.g. ‘I feel lightheaded after I exercise’) and reward (e.g. ‘I am allowed to eat more when I exercise’). However, this measure focuses on eating in general, rather than exclusively focusing on unhealthy eating, which might be motivated by different factors. Increased consumption of unhealthy foods and patterns of unhealthy eating are associated with numerous health risks (for review see Micha et al., 2017). Thus, given that compensatory eating of unhealthy food after exercise can be maladaptive for people who are exercising in order to reach specific health or weight-loss goals, it is important to identify the reasons why people might eat unhealthily post-exercise. It should also be noted that the structure of the CEMQ was not robust across samples, highlighting the need for further exploration of reasons underlying compensatory eating. Thus, the aim of the current research was to develop and validate a scale to assess people’s reasons for eating less healthily after exercise.

Based on the existing literature, we proposed five potential reasons that people might have for engaging in unhealthy compensatory eating after exercise:

### Reward for effort

People may eat unhealthily post-exercise because they feel that they deserve a reward for the effort they have put into exercising (Dohle, Wansink, & Zehnder, 2015; McCaig et al., 2016; Werle et al., 2015). McCaig et al. proposed that a reward explanation might explain why their participants ate more when they believed that they had burned more calories.

### Moral licensing

Moral Licensing refers to the finding in the prosocial behaviour literature that recalling a moral behaviour that one has previously completed (e.g. volunteer work, pro-environmental behaviour, helping a friend) reduces the likelihood of taking subsequent moral action (e.g. donating to charity; for review see Blanken, van de Ven, & Zeelenberg, 2015). Given that health behaviours are often viewed as having a moral component (e.g. viewing foods as ‘good’ foods and ‘bad’ foods), the Moral Licensing explanation might be applicable to compensatory eating after exercise (cf. Messner & Brügger, 2015). That is, exercise (a ‘good’ behaviour) might provide moral grounds for future indulgence (a ‘bad’ behaviour).

### Goal progress

When people feel as though they have done something that contributes to progress towards a specific goal (e.g. losing weight), they may be less likely to take subsequent action towards that goal. Recent research has shown that giving participants a weight-loss supplement (actually a placebo) compared to an openly administered placebo resulted in participants choosing less healthy foods in a buffet, choosing less healthy beverages, and consuming more hedonic food in a taste test, and that these choices were mediated by perceived progress towards a weight-loss goal (Chang & Chiou, 2014a, 2014b). Thus, if people feel as though they have progressed towards a health or weight goal by exercising, they may be subsequently less motivated to engage in future goal-directed actions like eating healthily.

### Reduced self-control

The strength model of self-control suggests that an individual’s capacity for self-control is a limited resource that, when depleted, can result in poorer subsequent self-control (Heatherton & Tice, 1994). In the context of eating behaviour, tasks that require self-control such as emotion suppression (e.g. asking people to watch an emotional video and suppress any emotional thoughts, feelings and facial expressions) can result in increased food intake in subsequent taste tests (e.g. Hofmann, Rauch, & Gawronski, 2007; Vohs & Heatherton, 2000). Therefore, if people require self-control to engage in exercise, then they may be giving in to tempting foods after exercising as a result of reduced self-control.

### Caloric compensation

People may eat more after exercising with the aim of ‘balancing out’ the calories burned during the exercise session. This mindset surrounding ‘balancing out’ calories by exercising (and ignoring the nutritional value of foods) is prevalent in current public health information and articles (e.g. ‘It takes 493 burpees to burn off a 100 g slice of chocolate mud cake’; Steen, 2017). Of course, the converse of this perspective is that, if people believe that doing extra exercise can compensate for unhealthy foods they have consumed, then they might similarly believe that exercising permits consumption of additional calories from unhealthy foods.

## Aim of the present research

The primary aim of the current research was to develop a measure that assesses reasons why people eat less healthily after they exercise. In Stage 1, potential items for the Compensatory Unhealthy Eating Scale (CUES) were subjected to exploratory factor analysis. Stage 2 sought to replicate the factor structure in a student sample using confirmatory factor analysis. Finally, in Stage 3, the factor structure was tested in another sample using confirmatory factor analysis. A secondary aim was evaluating the psychometric properties of the scale. Internal consistency and correlations between subscales were assessed in all stages, and criterion-related validity and construct validity were assessed in Stage 3.

## Stage 1: scale development

The purpose of Stage 1 was to develop a scale to measure reasons why people engage in compensatory eating behaviour after exercising. Potential items were generated by the researchers, rated by an online participant sample, and then subjected to exploratory factor analysis.

### Method

#### Participants

In Stage 1, participants (*N* = 490) were residents of the United States who were recruited online via the Amazon Mechanical Turk (MTurk) website and were reimbursed USD $1.20 for participating. See Table 1 for demographic data for the subset of participants that responded to the scale items (*n* = 443).

**Table 1.** Demographic characteristics for each of the 3 Stages, for those participants who responded to the CUES items.

	Stage 1	Stage 2	Stage 3
	(*n* = 443)	(*n* = 173)	(*n* = 198)
Gender (%)			
* Male*	51.24	25.43	43.94
* Female*	48.76	73.41	55.56
* Other*		1.16	0.50
Age (years)			
M *(SD)*	36.26 (11.31)	19.71 (3.48)	35.62 (11.51)
BMI			
M *(SD)*	26.82 (6.72)	21.82 (3.20)	26.47 (7.12)
Ethnicity (%)			
* Caucasian*	75.85	38.73	74.75
* Asian*	9.71	50.87	10.61
* African American*	7.22	-	7.07
* Latino/a*	5.87	-	3.03
* Alaska Native*	0.45	-	2.53
* Aboriginal/Pacific Islander*	-	0.58	-
* Other*	0.90	9.83	2.02
Weight goal (%)			
* Lose*	62.98	46.82	63.64
* No change*	31.15	36.99	27.78
* Gain*	5.87	16.18	8.59
Dieting status (%)			
* Dieting*	57.56	36.42	57.07
* Not dieting*	42.44	63.58	42.93
Exercise frequency			
M days/week (*SD)*	3.85 (1.64)	2.92 (1.51)	3.89 (1.64)
Exercise Intensity (%)			
* Vigorous*	28.22	36.47	31.40
* Moderate*	56.21	54.71	51.21
* Walking*	15.58	8.82	17.39

#### Item generation

A group of 10 health psychology researchers generated an initial pool of potential items for the CUES. Statements were generated for each of the five candidate reasons (Reward for Effort, Moral Licensing, Goal Progress, Reduced Self-Control, and Caloric Compensation) and then the lead researchers edited the statements for clarity and modified them such that began with the same stem: ‘After I’ve exercised, I sometimes eat less healthily because … ’. After editing, the refined item pool contained 39 items (see Table 2 for all items).

**Table 2.** Pattern matrix from Stage 1: 4 factor solution (principal axis factoring) for initial item pool of 39 items for the CUES.

	Factor			
	1	2	3	4
Eigenvalues (% Variance Explained)	6.05 (40.17)	4.72 (11.10)	2.65 (5.60)	1.27 (2.20)
Reward for Effort Items				
** I am rewarding myself for the effort I put in to exercise**	**0**.**912**	−0.037	0.032	0.055
** An indulgent treat is like a reward for good behaviour**	**0**.**901**	0.031	0.069	0.121
** I feel that I’ve earned a treat**	**0**.**938**	−0.039	−0.015	0.115
I feel like I deserve unhealthy foods more after I’ve put effort into a workout	0.579	0.205	−0.010	−0.119
treating myself to unhealthy food is okay if I’ve finished a hard workout	0.552	0.061	−0.050	−0.269
it is more appropriate to reward myself after putting effort into a workout	0.727	−0.053	0.028	−0.124
I feel like I can treat myself with food	0.749	0.060	0.050	−0.036
** I feel like I deserve a treat if I have done a lot of exercise**	**0**.**888**	−0.019	0.000	0.049
I like to give myself a food reward on days that I’ve put lots of effort into exercise	0.722	0.097	0.091	−0.011
the more effort I have put into my workout, the more I feel like I deserve a treat afterwards	0.806	0.039	0.005	0.000
Moral Licensing Items				
I am allowed to indulge in ‘naughty’ food if I’ve been virtuous and exercised	0.566	0.108	−0.101	−0.202
I don’t feel so bad about eating an unhealthy treat when I’ve worked out	0.580	−0.062	−0.137	−0.270
I don’t have to feel guilty about it if I’ve exercised	0.489	−0.017	−0.039	−0.332
I feel that it’s okay to be bad and indulge in unhealthy food	0.315	0.298	−0.068	−0.155
I can worry less about indulging if I’ve already exercised	0.398	0.019	−0.130	−0.464
I can have a treat without feeling guilty about it	0.654	−0.085	0.095	−0.085
Goal Progress Items				
I’ve already done something to improve my health	0.323	0.135	−0.191	−0.509
I’ve done something good for my health, so I’m less concerned about what I eat	0.320	0.207	−0.209	−0.419
I’ve already made a positive contribution to my health and wellbeing that day	0.355	0.079	−0.183	−0.518
I’ve already invested in my health that day	0.224	0.188	−0.164	−0.557
I’ve already done something beneficial for my health	0.320	0.140	−0.165	−0.504
I am less motivated to eat healthily	0.014	0.658	−0.096	−0.142
it balances out in terms of my overall health	0.143	0.218	−0.042	−0.481
Reduced Self-Control Items				
unhealthy food seems much more tempting	0.145	0.643	−0.053	0.083
I am often so tired that I can’t stop myself from eating unhealthy foods	0.070	0.693	0.232	0.120
I feel like I have less control over my diet	−0.116	0.751	0.045	0.020
** I don’t have as much self-control with regards to my food choices**	−0.023	**0**.**814**	0.000	0.010
I feel depleted and am unable to resist unhealthy foods	−0.002	0.753	0.183	0.084
** I find it hard to stop myself from eating something unhealthy**	0.045	**0**.**802**	−0.030	0.024
** I feel like I have less willpower to resist unhealthy food**	−0.092	**0**.**904**	−0.035	−0.045
** I feel like it is much harder to avoid unhealthy treats**	−0.021	**0**.**802**	0.045	−0.037
Caloric Compensation Items				
** my body needs the energy**	0.119	0.105	**0**.**662**	−0.055
** I’ve burned some extra calories**	0.068	−0.066	0.063	**−0**.**700**
** I need to refuel my body with calorie rich food**	0.089	0.120	**0**.**675**	−0.172
** I’ve burned lots of calories**	−0.032	−0.050	0.144	**−0**.**718**
I can afford to because I am in calorie deficit	−0.028	−0.015	0.208	−0.586
** exercising allows me to consume more calories**	−0.025	−0.038	0.214	**−0**.**647**
** I try to eat more calories on days that I’ve exercised**	0.005	0.056	**0**.**558**	−0.213
** I have already burned extra calories from the hard workout**	0.082	−0.031	0.116	**−0**.**684**

Note: Items in bold had the highest primary loadings on each of the four factors and were retained for the final scale. Item stem = ‘After I’ve exercised, I sometimes eat less healthily because … ’.

#### Procedure

The study was described as a study about ‘opinions on eating and exercise’. To be eligible for the study, participants had to report that they typically exercised at least once a week, but no other criteria about compensatory eating were specified so that recruitment was kept unbiased. After providing informed consent, participants were given a one-item measure of compensatory eating (How often do you eat less healthily you exercise? 1 = *never*; 7 = *always*). Participants who reported eating less healthily after exercise ‘never’ were then excluded (*n = *47), and the remaining participants (*n *= 443) were asked to respond to the 39 potential CUES items in random order.

The instructions for the CUES were as follows: ‘Below are some reasons why people might eat less healthily after they’ve exercised. Please rate the extent to which you agree that each reason applies to you.’ (1 = *strongly disagree*; 7 = *strongly agree*).

After responding to the items, participants then provided demographic information including age, gender, height and weight (used to calculate BMI), and ethnicity. They also reported how frequently they exercised (days per week), the intensity of their typical exercise (vigorous, moderate, or walking), whether they were currently dieting or watching what they ate (yes or no) and whether they were currently trying to gain or lose weight (want to lose weight, do not want to change weight, or want to gain weight).

The study protocol was approved by the university’s Human Research Advisory Panel (File 2972), and all participants provided informed consent.

### Results

#### Frequency of compensatory eating

The mean score on the frequency of compensatory eating item was 3.04 (*SD = *1.20). About one third (35.92%) of all participants (*N = *490) endorsed eating less healthily after they exercised at least ‘sometimes’ (i.e. ≥ 4; see online supplementary Table S1).

#### Exploratory factor analysis

Frequency distribution graphs for each item were inspected. Item responses for each item were approximately normally distributed. None of the items showed insufficient variation in the responses, with item responses spread across all seven answer options for all items, and no response option capturing more than 45% of all responses (cf. Dima, 2018). There was no missing data. No items were excluded on the basis of item distributions.

The 39 potential CUES items were subjected to factor analysis with principal axis factoring. Oblique rotation (direct oblimin) was chosen because it was expected that the resultant factors would be correlated. The sample size was good both according to recommendations for total sample size of 400–500 (Comfrey & Lee, 1992) and according to recommendations for at least a 10:1 participant:item ratio (Worthington & Whittaker, 2006). Indeed, the Kaiser-Meyer-Olkin (KMO) measure of sampling adequacy was .96, indicating that the sample size was ‘superb’ for this analysis (Hutcheson & Sofroniou, 1999). The anti-image matrices showed that the individual KMO values were all > .81, which is well above the acceptable limit of .50 (Kaiser, 1974). Bartlett’s test of sphericity was significant, χ^2^ (741) = 12664.96, *p* <.001, indicating that the correlations between items were sufficiently large for factor analysis (Field, 2009), and inspection of the correlation matrix revealed that none of the items had low correlations with all other items. The determinant of the matrix was < .00001, indicating potential multicollinearity; however, inspection of the correlation matrix showed that none of the items had very high correlations with one another (all below .80).

Several criteria were used to determine the appropriate number of factors to retain including the traditional methods of Kaiser’s criterion (1960) and Cattell’s scree plot (1966), as well as modern methods of Velicer’s Minimum Average Partial test (MAP; 1976), and Parallel Analysis (Horn, 1965) which are widely accepted as being more accurate (Osborne, 2014; Velicer, Eaton, & Fava, 2000). The analysis revealed that there were 4 factors with eigenvalues over Kaiser’s criterion of 1 that together explained 59.08% of the total variance (Kaiser, 1960, see Table 2 for eigenvalues). The MAP test was run using a programme developed by O’Connor (2000) and also suggested a 4 factor solution (see Supplementary Table S2). However, the scree plot had a clear point of inflexion at the 4th component, indicating a 3 component solution (Field, 2009, see Supplementary Figure S1). Parallel analysis (run using an online engine; Patil, Singh, Mishra, & Donavan, 2007) revealed that 3 eigenvalues were larger than the 95th percentile of the random eigenvalues, also suggesting a 3 factor solution (see Supplementary Table S3).

Given that the various different criteria were suggestive of a 3 or 4 factor solution, principal axis factoring was then run constraining the number of factors to 3 and 4 respectively, and the solutions were compared. Although we had proposed 5 potential reasons underlying compensatory eating, a 5 factor solution was not considered given the lack of statistical support across all criteria. Further, the potential reasons were highly theoretical because they were drawn in part from domains outside of health.

Inspection of the pattern matrices for the 3 and 4 factor solutions revealed that both solutions were very similar, with the exception that the 4 factor solution subdivided the Caloric Compensation items into two separate factors, which appeared to have good theoretical face validity (discussed further below). The 4 factor solution was retained given that it is preferable to specify too many factors over too few (and risk loss of important information; Hayton, Allen, & Scarpello, 2004; Zwick & Velicer, 1986). See Table 2 for factor loadings for the 4 factor solution, and see also Supplementary Table S4 for the 3 factor solution.

In the 4 factor solution, the Reward for Effort items all had primary loadings on Factor 1, with no cross-loadings on the remaining factors. The Moral Licensing items also loaded onto Factor 1, but less strongly, and two items cross-loaded onto other factors. Most of the Goal Progress items cross-loaded onto Factor 1 and Factor 4. The Reduced Self-Control items had primary loadings on Factor 2, with no cross-loadings. Three Caloric Compensation items had primary loadings on Factor 3, and the remaining five items had primary loadings on Factor 4. The Factor 3 items were specific to an active need to consume calories after exercise (e.g. ‘ … I need to refuel my body with calorie rich food’); the Factor 4 items were specific to exercise providing permission to consume more food (e.g. ‘ … I’ve burned some extra calories’).

#### Item reduction

The pattern matrix and item loadings were first used to remove poorly performing items that cross-loaded on more than one factor (*n *= 6) or had low primary loadings below |.5| (*n* = 2). Next, we retained the four items with the highest primary factor loadings on each of Factor 1, Factor 2 and Factor 4, and retained all three items loading on Factor 3, for a total of 15 items. The purpose of paring down the items was to keep the scale brief and practicable for use, and to ensure each subscale consisted of an approximately equal number of items. The retained items had corrected-item total correlations that were between .54 and .83, which is acceptable even by a conservative cut-off of .50 (cf. Ladhari, 2010). All items, if deleted, resulted in a reduction to Cronbach’s alpha, with the exception of one item loading on Factor 3 which increased Cronbach’s alpha if deleted; however, this item was retained because the subscale only had 3 items. Items loading on a given factor also appeared to have conceptual coherence. Thus, 15 items were retained for the scale.

The principal axis factoring with oblique rotation (direct oblimin) was repeated on the same sample with the revised item-set to ensure that the factor structure was not altered by the paring down of items. Factor loadings followed the same pattern as in the initial analysis, and the pattern matrix approximated simple structure with no-cross loading. Factor 1 represented Reward for Effort (rewarding oneself for effort put into exercise), Factor 2 represented Reduced Self-Control (finding it harder to resist unhealthy food after exercise), Factor 3 represented Need to Consume calories (actively eating more to gain energy and refuel the body after exercise), and Factor 4 represented Permission to Consume calories (burning extra calories through exercise permits greater consumption). See Table 3 for pattern matrix.

**Table 3.** Pattern matrix from Stage 1: factor analysis (principal axis factoring) for final 15 items for the CUES.

	Factor			
	1	2	3	4
Eigenvalues (% Variance Explained)	5.49 (34.49)	2.50 (14.53)	2.14 (11.84)	1.03 (4.53)
Reward for Effort Subscale (α = .92)				
I am rewarding myself for the effort I put in to exercise	**0**.**814**	−0.001	−0.014	−0.086
I feel that I’ve earned a treat	**0**.**897**	−0.009	−0.038	0.011
I feel like I deserve a treat if I have done a lot of exercise	**0**.**824**	0.004	−0.027	−0.051
an indulgent treat is like a reward for good behaviour	**0**.**858**	0.025	0.083	0.059
Reduced Self-Control Subscale (α = .90)				
I don’t have as much self-control with regards to my food choices	−0.019	**0**.**808**	−0.002	0.005
I find it hard to stop myself from eating something unhealthy	0.026	**0**.**865**	−0.060	−0.024
I feel like I have less willpower to resist unhealthy food	−0.041	**0**.**919**	−0.031	−0.020
I feel like it is much harder to avoid unhealthy treats	0.053	**0**.**725**	0.114	0.037
Need to Consume Subscale (α = .78)				
my body needs the energy	0.026	0.014	**0**.**745**	0.043
I need to refuel my body with calorie rich food	0.048	0.004	**0**.**865**	0.015
I try to eat more calories on days that I’ve exercised	−0.061	0.011	**0**.**565**	−0.130
Permission to Consume Subscale (α = .84)				
I’ve burned some extra calories	0.030	0.017	−0.110	**−0**.**854**
I’ve burned lots of calories	−0.026	−0.001	0.039	**−0**.**769**
exercising allows me to consume more calories	−0.008	0.001	0.132	**−0**.**610**
I have already burned extra calories from the hard workout	0.092	0.001	0.013	**−0**.**724**

#### Internal consistency

Reliability analyses showed that all 4 subscales had at least acceptable internal consistency. Cronbach’s alpha was excellent for Reward for Effort (.92) and Reduced Self-Control (.90), good for Permission to Consume (.84) and acceptable for Need to Consume (.78), according to George and Mallery’s (2003) rules of thumb.

#### Correlations between subscales

All 4 subscales were significantly correlated with one another (*p*s < .010), indicating oblique rotation was appropriate. The Reward for Effort and Permission to Consume subscales were most highly correlated (*r = *.49, *p* < .001), and the lowest correlation was between the Reward for Effort subscale and the Need to Consume subscale (*r = *.15, *p* = .002)*.* See Table 4 for all subscale correlations.

**Table 4.** Correlations between subscales of the CUES in each of the three Stages.

	Stage 1 (*n* = 443)			Stage 2 (*n* = 173)			Stage 3 (*n* = 198)		
	Control	Need	Permission	Control	Need	Permission	Control	Need	Permission
Reward	.354**	.145**	.491**	.266**	.193*	.607**	.223**	.061	.409**
Control		.267**	.175**		.172*	.186*		.167*	.150*
Need			.399**			.476**			.438**

Note: Reward = Reward for Effort, Control = Reduced Self-Control, Need = Need to Consume, Permission = Permission to Consume.

*p < .05, **p < .01.

### Discussion

The aim of Stage 1 was to develop a scale to assess reasons why people might eat less healthily after they have exercised. The exploratory factor analysis resulted in a 15-item CUES with four subscales: Reward for Effort, Reduced Self-Control, Need to Consume and Permission to Consume. All the subscales were significantly correlated and had high internal consistency. Of the original five mechanisms that we proposed might underlie compensatory eating, Reward for Effort and Reduced Self-Control emerged as distinct subscales. The Caloric Compensation mechanism that we proposed was split into two new subscales: Need to Consume, which reflected an active seeking of extra calories after exercise, and Permission to Consume, which reflected the idea that exercising allows or permits the consumption of additional calories. The final two mechanisms that we proposed, Moral Licensing and Goal Progress, did not emerge as distinct subscales.

In Stage 2, we attempted to test the factor structure in a different sample using Confirmatory Factor Analysis. The first sample consisted of United States residents whereas the second sample were Australian students.

## Stage 2: scale replication

### Method

#### Participants

Participants (*N *= 180) were undergraduate psychology students at an Australian university who participated in the online study for course credit. See Table 1 for demographic data for the subset of participants that responded to the scale items (*n* = 173).

#### Procedure

All aspects of the procedure were very similar to Stage 1. As in Stage 1, participants reported how frequently they ate less healthily after they exercised on a 7-point scale anchored 1 = *never* and 7 = *always*. Participants who reported eating less healthily after exercise ‘never’ were then excluded (*n* = 7), and the remaining participants (*n* = 173) were asked to respond to the 15-item CUES developed in Stage 1, before completing the demographic questions. As in Stage 1, the study protocol was approved by the university’s Human Research Advisory Panel (File 2972), and all participants provided informed consent.

### Results

#### Frequency of compensatory eating

The mean score on the frequency of compensatory eating measure was 3.44 (*SD = *1.07). About half (50.55%) of all participants (*N* = 180) endorsed eating less healthily after they exercised at least ‘sometimes’ (i.e. ≥ 4; see online supplementary Table S1).

#### Confirmatory factor analysis

Confirmatory factor analysis using maximum likelihood estimation was carried out with the programme AMOS to examine whether the 4 factor structure developed in the first sample was replicated, and to assess the fit of the model. There was no missing data, and no items had insufficient variation in the responses.

All of the individual parameter estimates were statistically significant, and loadings ranged between .65 and .86 (see Figure S2). A range of goodness-of-fit statistics were used to evaluate the model fit as recommended (Shek & Yu, 2014). The chi-squared test was significant, χ^2^ (84) = 147.21, *p* <.001, indicating poor model fit, but the chi-squared test is strongly influenced by sample size. The ratio of χ^2^ to degrees of freedom was < 2, indicating good model fit (CMIN/df = 1.75) according to Schreiber and colleagues (Schreiber, Nora, Stage, Barlow, & King, 2006). According to Shek and Yu (2014), good model fit is indicated by comparative fit index (CFI), Tucker-Lewis’s index of fit (TLI), and normed fit index (NFI) > 0.90, and root mean square of approximation (RMSEA) < .10. For this model, CFI = 0.95, TLI = 0.94, NFI = 0.90, and RMSEA = 0.07, indicating reasonably good fit. However, the fit indices fall slightly below a more conservative threshold of 0.95 for TLI, and NFI, and RMSEA < 0.060 (Schreiber et al., 2006). Schreiber et al. (2006) also suggest the incremental fit index (IFI) > .95 is indicative of good fit, and IFI = .95 for this model. An examination of the modification indices revealed that none of the covariances or regression weights were unreasonably large (> 80 and > 50, respectively), indicating no misspecficiation in the model (Shek & Yu, 2014). Overall, the model fit appeared acceptable to good.

#### Internal consistency

Reliability analyses showed that all 4 subscales had at least acceptable internal consistency according to George and Mallery’s (2003) rules of thumb. Cronbach’s alpha was excellent for Reward for Effort (.91), good for Reduced Self-Control (.87) and Permission to Consume (.82) and acceptable for Need to Consume (.78),

#### Correlations between subscales

As in Stage 1, all 4 subscales were significantly positively correlated with one another (all *p*s < .050); see Table 4.

### Discussion

In Stage 2, we replicated the CUES in new sample. Confirmatory factor analysis revealed acceptable to good model fit. As with Stage 1, all 4 subscales had high internal consistency and were significantly and positively correlated with one another. The aim of Stage 3 was to further validate the scale by assessing construct validity and criterion validity. A new sample of American residents rated the scale items and completed several other measures pertaining to eating behaviour, exercise behaviour and compensatory eating which were used for the validity analyses.

## Stage 3: scale validation

### Method

#### Participants

Participants (*N *= 209) were American residents recruited via MTurk and were reimbursed USD $2.00 for participating. See Table 1 for demographic data for the subset of participants that responded to the scale items (*n* = 198).

#### Procedure

As in Stage 1 and 2, participants reported how frequently they ate less healthily after they exercised on a 7-point scale anchored 1 = *never* and 7 = *always*. Participants who reported eating less healthily after exercise ‘never’ were then excluded (*n = *11), and the remaining participants (*n = *198) were asked to respond to the 15-item CUES. Participants also completed a number of additional validation scales (described below) before providing demographic information. As the previous stages, the study protocol was approved by the university’s Human Research Advisory Panel (File 2972), and all participants provided informed consent.

#### Validation scales

##### Compensatory eating motivations questionnaire (CEMQ)

The CEMQ measures reasons why people engage in compensatory eating following exercise in general, rather than specifying unhealthy eating (Moshier et al., 2016). Given that the themes of the CEMQ are similar to those in our scale, we hypothesised that our Reward for Effort subscale would be positively correlated with CEMQ-reward and that our Need to Consume subscale would be positively correlated with CEMQ-recovery.

##### Compensatory health beliefs scale (CHBS)

This scale measures the degree to which people hold compensatory beliefs about how one poor health behaviour can be made up for (i.e. compensated for) by another beneficial behaviour in four different domains (substance use, eating/sleeping habits, stress and weight regulation; Knäuper et al., 2004). We hypothesised that our Permission to Consume subscale would be positively correlated with the CHBS because having stronger beliefs about compensation is likely to permit compensatory eating.

##### Three factor eating questionnaire (TFEQ)

The TFEQ is a 51-item scale that measures three dimensions of disordered eating tendencies: Restraint, Hunger, and Disinhibition (Stunkard & Messick, 1985). We hypothesised that TFEQ Disinhibition would be positively correlated with the Reward for Effort and Reduced Self-Control subscales because disinhibited eaters are more likely to eat in response to external factors. We also hypothesised that TFEQ Restraint would be positively correlated with Permission to Consume because restrained eaters are more likely to be monitoring their food intake and choosing foods based on caloric values.

##### International physical activity questionnaire (IPAQ)

The IPAQ is a measure of amount of physical activity that captures both duration and intensity of exercise carried out in the last seven days (Craig et al., 2003). The number of minutes spent doing each intensity of physical activity is multiplied by a metabolic equivalent (MET) score for that activity to generate a total score of the number of metabolic equivalent minutes (MET-minutes) expended during exercise. We expected MET-minutes to be positively correlated with the Need to Consume subscale because people who are more physically active may feel a greater need to refuel after exercising.

##### Reasons for exercise inventory (REI)

The REI measures various different reasons why people might engage in exercise (Silberstein, Striegel-Moore, Timko, & Rodin, 1988). Following previous research (e.g. Strelan, Mehaffey, & Tiggemann, 2003; Vartanian, Wharton, & Green, 2012), we grouped the seven subscales into two overarching categories: appearance reasons (composed of weight control, attractiveness and tone subscales) and health reasons (composed of fitness, mood, health and enjoyment subscales). We hypothesised that our Need to Consume subscale would be positively correlated with REI-health reasons for exercise because people who exercise for health reasons may be more likely to want to refuel their bodies after exercise, but we had no specific hypotheses regarding REI-appearance reasons.

### Results

#### Frequency of compensatory eating

The mean score on the frequency of compensatory eating measure was 3.51 (*SD = *1.17). Just over half (55.68%) of all participants (*N* = 209) endorsed eating less healthily after they exercised at least ‘sometimes’ (i.e. ≥ 4; see online supplementary Table S1).

#### Confirmatory factor analysis

As in Stage 2, Confirmatory factor analysis using maximum likelihood estimation was carried out with the programme AMOS to examine whether the CUES structure replicated. There was no missing data, and no items had insufficient variation in the responses.

All of the individual parameter estimates were statistically significant, and loadings ranged between .64 and .91 (see Figure S3). The chi-squared test was significant, χ^2^ (84) = 128.68, *p *= .001, indicating poor model fit; however, the ratio of χ^2^ to degrees of freedom was < 2, indicating good model fit (CMIN/df = 1.53). The values for CFI, TLI and IFI were all > 0.95, indicating good model fit and NFI was > 0.90 indicating reasonably good fit (CFI = 0.98, TLI = 0.97, IFI = 0.98, NFI = 0.93). RMSEA = 0.52, indicating good fit (Schreiber et al., 2006). An examination of the modification indices revealed that none of the covariances or regression weights were unreasonably large (> 80 and > 50, respectively), indicating no misspecfication in the model (Shek & Yu, 2014). Overall, the model fit appeared good.

#### Internal consistency

Reliability analyses showed that all 4 subscales had good-excellent internal consistency according to George and Mallery’s (2003) rules of thumb. Cronbach’s alpha was excellent for Reward for Effort (.91), Reduced Self-Control (.90), Permission to Consume (.90) and good for Need to Consume (.80),

#### Correlations between subscales

All of the subscales were significantly positively correlated with one another (*p*s < .050) with the exception of a nonsignificant correlation between the Reward for Effort and Need to Consume subscales, *r* = .06, *p* = .395. The remaining correlations between the subscales were of similar magnitude to the first two Stages (see Table 4).

#### Criterion-related validity

Concurrent criterion-related validity was assessed in Stage 3 by correlating the mean scores for each of the CUES subscales with the measure of self-reported compensation frequency. As expected, compensation frequency was significantly positively correlated with all four subscales (Reward for Effort: *r *= .30, *p *< .001; Reduced Self-Control: *r *= .41, *p *< .001; Need to Consume: *r *= .23, *p *= .001; Permission to Consume: *r *= .27, *p* < .001).

#### Construct validity

Convergent construct validity was assessed by examining the correlations between the four subscales of the CUES and other relevant measures of eating and exercise behaviour (see Table 5 for all correlations).

**Table 5.** Correlations between CUES subscales and other measures included in Stage 3.

	Reward for Effort	Reduced Self-Control	Need to Consume	Permission to Consume
CEMQ				
Reward	.599**	.296**	.316**	.474**
Relief	.049	.326**	.198**	.091
Recovery	.007	.045	.523**	.261**
CHBS	.153*	.361**	.087	.157*
TFEQ				
Restraint	−.002	.072	.037	.181*
Disinhibition	.198**	.509**	−.012	.188**
Hunger	.203**	.437**	.099	.192**
IPAQ-METS	−.032	−.046	.229**	.090
REI				
Health	.106	- .011	.220**	.112
Appearance	.144*	.294**	.085	.241**

Note: CEMQ = Compensatory Eating Motivations Questionnaire, CHBS = Compensatory Health Beliefs Scale, TFEQ = Three Factor Eating Questionnaire, IPAQ-METS = International Physical Activity Questionnaire Metabolic Equivalent Minutes, REI = Reasons for Exercise Inventory.

*p < .05, **p < .01.

##### Compensatory eating motivations questionnaire (CEMQ)

As hypothesised, CEMQ-reward was correlated most strongly with our Reward for Effort subscale (*r *= .60, *p *< .001), but it was also correlated the other three subscales. Also as expected, CEMQ-recovery subscale was most strongly correlated with the Need to Consume subscale (*r* = .52, *p* < .001), but it was also correlated with Permission to Consume (*r* = .26, *p* < .001). We had no specific hypotheses about the relief subscale, but found that CEMQ-relief was positively correlated with both Reduced Self-Control (*r* = .34 *p* < .001) and Need to Consume (*r* = .20, *p* = .005), but not with the other two subscales.

##### Compensatory health beliefs scale (CHBS)

As expected, the CHBS was significantly positively correlated with Permission to Consume (*r* = .16, *p *= .027). However, it was also positively correlated with Reward for Effort (*r* = .15, *p *= .032) and Reduced Self-Control (*r* = .36, *p *< .001).

##### Three factor eating questionnaire (TFEQ)

As expected, TFEQ-disinhibition was positively correlated with Reward for Effort (*r* = .20, *p *= .005) and Reduced Self-Control (*r* = .51, *p *< .001), but it was also correlated with Permission to Consume (*r* = .19, *p *= .008). Also as hypothesised, the TFEQ-restraint subscale was positively correlated with Permission to Consume (*r* = .18, *p *= .011), and not any of the remaining three subscales. We had no specific hypotheses regarding the TFEQ-hunger scale, but found that TFEQ-hunger was correlated with Reward for Effort (*r* = .20, *p *= .004), Reduced Self Control (*r* = .44, *p *< .001), and Permission to Consume (*r* = .19, *p *= .007), but not Need to Consume.

##### International physical activity questionnaire (IPAQ)

As expected, MET-minutes were significantly positively correlated with the Need to Consume (*r* = .23, *p *= .001), but not the remaining subscales.

##### Reasons for exercise inventory (REI)

As predicted, REI-health reasons for exercise was positively correlated with Need to Consume (*r* = .22, *p *= .002), but not the other three subscales. The opposite pattern was seen for REI-appearance reasons, which was positively correlated with Reward for Effort (*r* = .14, *p *= .043), Reduced Self-Control (*r* = .29, *p *< .001) and Permission to Consume (*r* = .24, *p *= .001), but not Need to Consume.

### Discussion

In Stage 3, we replicated the CUES in a new sample. Confirmatory factor analysis revealed good model fit and, as with the previous stages, the subscales had good internal consistency. The subscales were all correlated with measures of the frequency of compensatory eating, indicating good criterion validity. The subscales were also correlated with related measures, indicating good construct validity.

## General discussion

The aim of the present research was to develop and validate a scale to measure reasons why people might eat less healthily after they exercise. We had initially proposed five potential reasons for compensatory eating based on the existing literature: Reward for Effort, Moral Licensing, Goal Progress, Reduced Self-control, and Caloric Compensation. The resultant scale that emerged after factor analysis was a 15-item measure with four subscales. Two of the subscales (Reward for Effort and Reduced Self-Control) were in line with our initial proposal. However, the items that were thought to pertain to a Caloric Compensation explanation loaded onto two separate factors, revealing that this construct may be more nuanced than originally assumed. These new factors were comprehensible and conceptually distinct and became the Need to Consume and Permission to Consume subscales. The remaining two proposed reasons (Moral Licensing and Goal Progress) loaded onto Reward for Effort subscale, but much less strongly, and thus were not part of the final scale. The four subscale factor structure was replicated across all three Stages, and all of the final subscales had good face validity, conceptual clarity and internal consistency. The subscales are described in more detail below.

### CUES subscales

#### Reward for effort

The Reward for Effort subscale reflects the tendency to eat less healthily after exercise in order to treat oneself. This reason for compensatory eating is consistent with previous research which showed that feeling as though one has put more effort into exercise (i.e. burned more calories) results in greater subsequent food intake (McCaig et al., 2016). Reward for Effort was positively correlated with CEMQ-reward, which was expected given that both measures assess the construct of food as a reward, and Reward for Effort was also positively correlated with the CHBS, a general measure of compensatory beliefs. In addition, Reward for Effort was positively correlated with TFEQ-disinhibition and hunger, suggesting that those with disordered eating tendencies might also use reward as a justification for eating less healthily after exercise. Finally, Reward for Effort was positively correlated with appearance reasons for exercise.

#### Reduced self-control

The Reduced Self-Control subscale reflects feeling as though one has less willpower to control one’s food choices after having exercised. Like the Reward for Effort subscale, the Reduced Self-control subscale was also positively correlated with the CHBS, CEMQ-reward, TFEQ-disinhibition and TFEQ-hunger, and appearance reasons for exercise, which again might suggest this reason is common amongst people who are likely to be dieting or who have disordered eating tendencies. Reduced Self-control was also positively correlated with CEMQ-relief, which suggests that people who find exercise more aversive might also feel they have less self-control following exercise.

#### Need to consume

The Need to Consume subscale reflects an active desire to eat more calories after exercising to refuel the body out of necessity. The Need to Consume subscale had a strong positive correlation with CEMQ-recovery, which was expected given the conceptual similarity of the items within these subscales. Need to Consume also had smaller positive correlations with CEMQ-reward and CEMQ-relief. Also as hypothesised, Need to Consume was positively correlated with health reasons for exercise and with IPAQ-METS, which suggests that health-focused exercisers and more frequent exercisers respectively might be more likely to cite Need to Consume reasons.

#### Permission to consume

The Permission to Consume subscale reflects the idea that the caloric deficit created through exercising permits or allows one to eat less healthily and consume extra calories. In contrast to the Need to Consume subscale, which is about needing to eat more calories to recover from exercise, Permission to Consume reflects the idea that it is permissible (but not necessary) to eat more after exercise. Both the Need to Consume and Permission to Consume subscales were positively correlated with CEMQ-reward and CEMQ-recovery. However, Permission to Consume was also positively correlated with appearance reasons for exercise, while Need to Consume was correlated with health reasons for exercise. In addition, Permission to Consume (but not Need to Consume) was also positively correlated with TFEQ-restraint, disinhibition, and hunger, which suggests that people with disordered eating tendencies are more likely to explain their compensatory eating in terms of exercise permitting greater caloric intake. Thus, although Permission to Consume and Need to Consume are both calorie-centric, Permission appears to reflect more of a permissive, maladaptive style of compensation whereby exercise permits caloric indulgence, whereas Need reflects more of an intentional health-focused style of compensation whereby exercise requires caloric balancing.

### Similarities and distinctions between CUES subscales

One consistent finding from the present research was that Reward for Effort and Permission to Consume were the most highly correlated of all the pairs of subscales (*r*s ranged from .41–.61). Conceptually, these two subscales differ in that Permission to Consume items are about exercise permitting consumption of more calories, whereas the Reward for Effort items do not refer to calories and instead appear to reflect treating oneself for effort. However, the strong correlation between subscales could indicate that reward and caloric compensation beliefs tend to co-occur even though they are different from one another.

Another point of interest is the clear distinction that emerged between Need to Consume subscale and the other three subscales, both conceptually, and in terms of correlations with other scales. As stated earlier, the Need to Consume subscale was positively correlated with health reasons for exercise, but it was uncorrelated with appearance reasons for exercise, the CHBS, and the subscales of the TFEQ measuring dimensions of eating behaviour. The opposite pattern was observed for the Reward for Effort, Reduced Self-Control and Permission to Consume: they were positively correlated with appearance reasons for exercise, the CHBS, and some or all of the TFEQ subscales, but uncorrelated with health reasons for exercise. Given that endorsement of appearance-related reasons for eating is associated with negative outcomes such as body image concerns (e.g. Vartanian et al., 2012) and that high levels of TFEQ-restraint, disinhibition and hunger are also associated with negative outcomes such as weight dissatisfaction (e.g. Bond, McDowell, & Wilkinson, 2001), compensatory eating that reflects Reward for Effort, Reduced Self-Control and Permission to Consume may be indicative of an unhealthy behavioural pattern. It is possible that compensatory eating for these reasons, in particular, might be problematic for people trying to achieve specific health and weight-loss goals. In contrast, Need to Consume appears reflect more of a fitness orientation to exercise.

In Stage 1, a surprising finding was that the items pertaining to two of the originally proposed reasons (Moral Licensing and Goal Progress) loaded onto the same factor as the Reward for Effort items, albeit less strongly. Indeed, all three concepts are similarly focused on the idea that doing something ‘good’ seems to permit one to then behave oppositely, whether this ‘good’ initial action be something effortful (i.e. Reward for Effort), something virtuous (i.e. Moral Licensing) or something that moves one closer to one’s goals (i.e. Goal Progress). It may be that these latter explanations are not distinguishable from Reward for Effort. Perhaps these concepts are actually largely overlapping but happen to be described with different terminology simply because they emerged from different literatures: Reward originates from the consumer choice literature (e.g. Khan & Dhar, 2006), Moral Licensing is derived from the prosocial behaviour literature (see Blanken et al., 2015), and Goal Progress has been proposed within the health literature (e.g. Chang & Chiou, 2014a, 2014b; Hennecke & Freund, 2014). Alternatively, it is also possible that these three reasons are conceptually different, but that the generated items were not able to adequately capture the subtle distinctions. Understanding the conceptual similarity and differences among these constructs would be an important avenue for future research. For example, experimental studies involving exercise and subsequent eating could explore whether framing a food item as a reward for effort vs. a reward for ‘being good’ and exercising vs. a reward for making progress towards health have similar effects on subsequent intake. It might also be informative to investigate whether individual differences help distinguish these constructs. For example, someone high in religiosity might find moral licensing resonates with them more strongly, whereas goal progress might be more relevant to people with high health motivation or specific health goals.

### Application, limitations and future directions

The CUES has potential as a tool for researchers wanting to assess reasons underlying compensatory eating behaviour, specifically with respect to eating less healthily after exercise. The CUES might also be beneficial for use with populations trying to achieve certain health or weight loss goals, in that clinicians may be able to use this scale to identify (and then challenge) relevant maladaptive compensatory beliefs. A strength of the CUES is the robustness of the factor structure across various different samples. Specifically, we recruited two American samples that were predominately Caucasian, and a sample of Australian students that were predominately Asian. Across Stages the samples also had good representation with regards to gender, age and BMI. The scale therefore appears to have considerable generalisability to different populations. However, it is worth noting that there were also some similarities between samples including that the majority of participants wanted to lose weight and were currently dieting. Future research could explore the extent of the generalisability of this measure in more diverse samples.

The development of the CUES has provided numerous useful insights into understanding compensatory eating, but further work is needed to elucidate who is most likely to engage in this behaviour, and under what circumstances. One limitation and cautionary note for the CUES is that, as yet, the scale has not been tested to determine whether it can explain actual compensatory eating behaviour. Although we found that CUES mean subscale scores correlated with self-reported frequency of compensation and other measures of related behaviours, experimental research is needed to test the concurrent and predictive validity of the scale. Another limitation is that some of the final subscales contained items that are somewhat similar, which might suggest that there is some redundancy between these items. It would be important for future research to explore whether these nuances are meaningful and refine the scale if needed, for example, using item response theory. In addition, cognitive interviews or other qualitative approaches might be useful in deepening current understanding of reasons underlying compensatory eating.

Future research is also needed to assess whether different reasons for compensatory eating might apply more to some groups of people than others. Further, perhaps certain situations might be more likely to elicit compensation for one particular reason compared to another. One way to examine the contributing roles of individual difference and contextual factors may be through the use of ecological momentary assessment (Stone & Shiffman, 1994). This type of study would allow researchers to examine compensatory behaviour in much greater detail as it unfolds over time, and gain a better understanding of whether different people tend to favour a certain explanation for compensation, and whether they tend to use different explanations in different circumstances.

### Conclusion

The present research demonstrated the reliability and validity of a 15-item scale to measure reasons for eating less healthily after exercise. Across all three samples, the CUES had a clean factor structure that was face-valid and had good internal consistency. Convergent validity of the subscales was demonstrated in Stage 3. Further research should examine who is most likely to compensate and under what circumstances. Broadening current knowledge of compensatory eating after exercise has the potential to facilitate development of strategies to improve health behaviour regulation.
